# Early ERK1/2 activation promotes DRP1-dependent mitochondrial fission necessary for cell reprogramming

**DOI:** 10.1038/ncomms11124

**Published:** 2016-03-31

**Authors:** Javier Prieto, Marian León, Xavier Ponsoda, Ramón Sendra, Roque Bort, Raquel Ferrer-Lorente, Angel Raya, Carlos López-García, Josema Torres

**Affiliations:** 1 Departamento de Biología Celular, Universidad de Valencia, Burjassot 46100, Spain; 2 Departamento de Bioquímica y Biología Molecular, Universidad de Valencia, Burjassot 46100, Spain; 3 Unidad de Hepatología Experimental, CIBERehd, Instituto de Investigación Sanitaria La Fe, Valencia 46026, Spain; 4 Centre de Medicina Regenerativa de Barcelona, Barcelona 08003, Spain; 5 Centro de Investigación Biomédica en Red en Bioingeniería, Biomateriales y Nanomedicina, Madrid 28029, Spain; 6 Institució Catalana de Recerca i Estudis Avançats, Barcelona 08010, Spain

## Abstract

During the process of reprogramming to induced pluripotent stem (iPS) cells, somatic cells switch from oxidative to glycolytic metabolism, a transition associated with profound mitochondrial reorganization. Neither the importance of mitochondrial remodelling for cell reprogramming, nor the molecular mechanisms controlling this process are well understood. Here, we show that an early wave of mitochondrial fragmentation occurs upon expression of reprogramming factors. Reprogramming-induced mitochondrial fission is associated with a minor decrease in mitochondrial mass but not with mitophagy. The pro-fission factor Drp1 is phosphorylated early in reprogramming, and its knockdown and inhibition impairs both mitochondrial fragmentation and generation of iPS cell colonies. Drp1 phosphorylation depends on Erk activation in early reprogramming, which occurs, at least in part, due to downregulation of the MAP kinase phosphatase Dusp6. Taken together, our data indicate that mitochondrial fission controlled by an Erk-Drp1 axis constitutes an early and necessary step in the reprogramming process to pluripotency.

## Abstract (web summary)

Reprogramming of somatic cells is a stepwise process where cells must overcome several barriers before reaching the pluripotent state. Here the authors show that mitochondrial fission in response to ERK1/2 signalling is an important early step during reprogramming to pluripotency.

## Graphical abstract

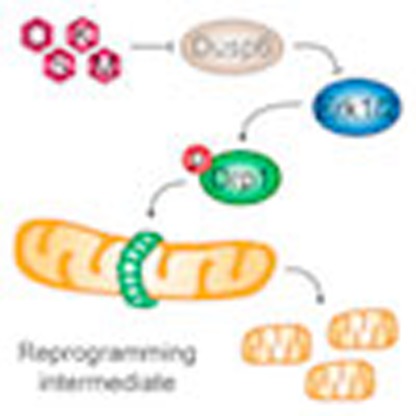

Somatic cells can be reprogrammed to induced pluripotent stem (iPS) cells by forced expression of Oct4 (also known as Pouf5.1), Sox2, Klf4 and c-Myc^1^,^2^ (named as OSKM hereinafter). Reprogramming of somatic cells is a stepwise process and cells must overcome several barriers before reaching the pluripotent state^3^,^4^. These barriers include the downregulation of somatic gene expression, senescence and acquisition of epithelial-like features during the early and middle steps, and stabilization of the pluripotent state by inducing a robust activation of the gene expression network characteristic of undifferentiated embryonic stem (ES) cells in later stages. Epigenetic remodelling of the somatic genomic landscape occurs throughout the reprogramming process leading to the bivalent state of chromatin representative of ES cells^5^.

Mitochondria are key organelles for cellular homeostasis that join by fusion and divide by fission^6^. Fusion is mediated by Mitofusin-1 and -2 (Mfn1 and Mfn2) and optic atrophy 1 (Opa1) proteins located at the outer and inner mitochondrial membranes, respectively. Fission is mediated by dynamin-related protein 1 (Drp1) (ref. 7), a cytosolic protein that, on activation, is recruited to the surface of mitochondria with the collaboration of accessory proteins, such mitochondrial fission factor (Mff), mitochondrial fission protein 1 (Fis1) or mitochondrial elongation factor 1 and 2 (Mief1/MiD51 and Mief2/MiD49, respectively)^8^,^9^,^10^,^11^,^12^,^13^,^14^. Mitochondrial fission starts by the formation of an initial constriction in the mitochondria at contact sites with the endoplasmic reticulum^15^,^16^. Activated Drp1 is then recruited to the constricted mitochondrial membrane, forming a ring that eventually fragments mitochondria in a GTPase-dependent manner^17^.

Drp1 undergoes post-traslational modifications that regulate its recruitment to mitochondria in different settings, including phosphorylation, S-nitrosilation, ubiquitination and sumoylation^6^,^18^. For instance, mitochondrial association is promoted by phosphorylation of human DRP1 at serine 616 (S616) by CDK1 during mitosis or by PKCδ in neuronal cells under oxidative stress^19^,^20^. On another hand, phosphorylation of serine 637 in human DRP1 by PKA impairs its recruitment to mitochondria, whereas dephosphorylation of this residue by PP2A or calcineurin favours its recruitment to these organelles^21^,^22^,^23^. In addition, ROCK1-, CaMKIα- and AMPK-mediated phosphorylation of serine 637, and GSK3β phosphorylation of serine 693 in DRP1 play important roles in the regulation of DRP1 recruitment to the mitochondria in different cellular contexts^24^,^25^,^26^,^27^.

Recently, it was shown that phosphorylation of DRP1-S616 by ERK2 promotes mitochondrial fission and mitogen-activated protein kinase (MAPK)-driven tumour growth^28^,^29^. MAPKs are a highly relevant family of protein kinases, which play a major role in maintaining cellular homeostasis in response to external and intracellular cues as well as in the regulation of a wide range of physiological processes. The activation status of MAPKs reflects the balance of their phosphorylation by specific MAPK-kinases and their dephosphorylation by inactivating serine/threonine phosphatases, dual specificity protein phosphatases (DUSPs) and/or protein tyrosine phosphatases (PTPs). Among these, the DUSP family of protein phosphatases is dedicated to the specific regulation of MAPKs in mammalian cells, with DUSP6, DUSP7 and DUSP9 being the major cytoplasmic phosphatases that dephosphorylate and inactivate ERK1/2 (refs 30, 31).

Although recent reports have highlighted the importance of mitochondria in cell reprogramming from a metabolic perspective^32^,^33^,^34^, the role of the dynamics of these organelles in this process remains unexplored. Here we show that expression of reprogramming factors downregulates *Dusp6* gene expression to activate ERK signalling and promote a Drp1-dependent mitochondrial fission pathway that is necessary for cell reprogramming to pluripotency.

## Results

### Cell reprogramming induces mitochondrial fission

**Figure 1:**
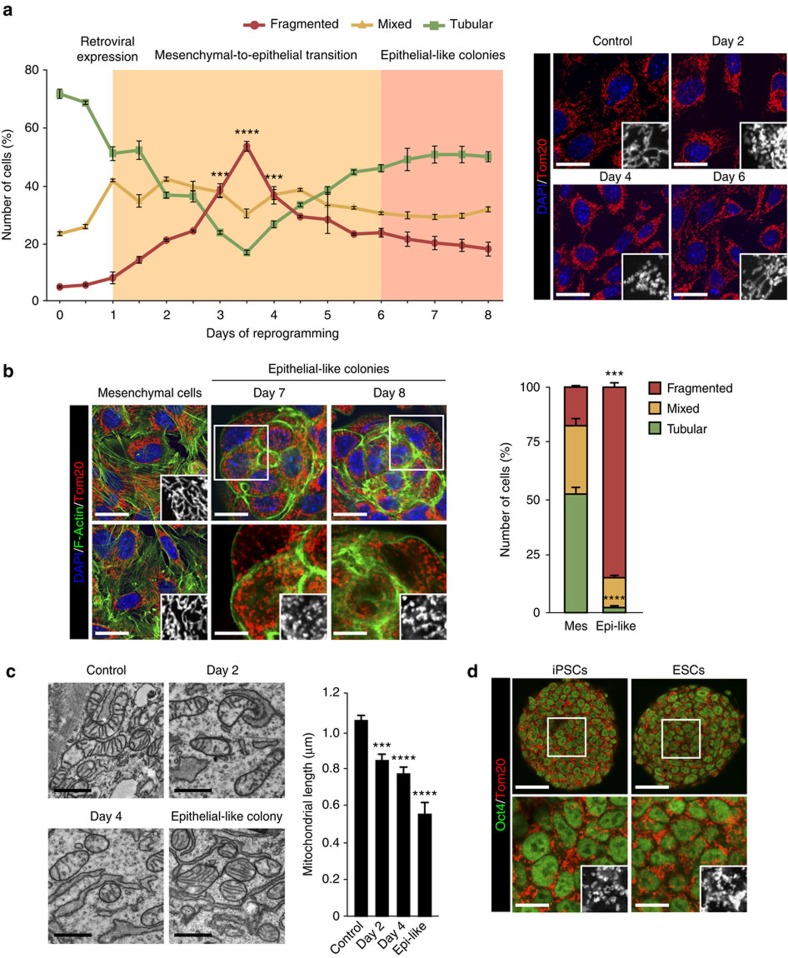
Cell reprogramming induces mitochondrial fission. (**a**) MEFs were mock-infected (day 0, control) or OSKM-transduced. At the indicated days, cells were fixed and mitochondrial morphology assessed by IF. (right) IF images of MEFs stained with anti-Tom20 antibody (red) before (control) or after expressing the OSKM factors for the indicated days. Insets show a black and white magnification of the pictures. DAPI (blue) was used as a nuclear counterstaining. Scale bar, 24 μm. Graph on the left, quantification of the different mitochondrial morphologies observed in MEFs before (day 0) or at the indicated days after OSKM expression (*n*=3). Shaded areas depict the timing for MET (orange) and the appearing of epithelial-like colonies (pink). (**b**) IF images of mesenchymal cells (scale bar, 24 μm (leftmost)) or epithelial-like colonies (scale bar, 16 μm (middle); scale bar, 16 μm (right)) found in the cultures at the indicated days of reprogramming showing F-actin and mitochondria stainings. Middle and right lower images are a magnification of the indicated area in the respective upper panels. Scale bar, 8 μm. Insets show a black and white magnification of the pictures. DAPI (blue) was used as a nuclear counterstaining. Graph on the right, quantification of the indicated mitochondrial morphologies observed in the mesenchymal cells (Mes) or epithelial-like colonies (Epi-like) at day 8 of reprogramming (*n*=3). (**c**) TEM micrographs of MEFs before (control) or at the indicated days after OSKM expression, or epithelial-like colony (Epi-like) at day eight of reprogramming, displaying the ultrastructural characteristics of their mitochondria. Scale bar, 800 nm. Graph on the right, quantification of the mitochondrial length observed in the indicated cells (*n*⩾800). (**d**) IF images of pluripotent cells stained with anti-Oct4 (green) and anti-Tom20 antibodies (red). Insets below are a black and white magnification of the pictures illustrating mitochondrial morphologies. Scale bar, 40 μm (upper); scale bar, 16 μm (lower), respectively. Data are represented as mean±s.e.m.(*P<0.05, **P<0.01, ***P<0.001, ****P<0.0001). One-tailed unpaired Student's *t*-test was used to compare data sets.

We sought to investigate mitochondrial dynamics during cell reprogramming. Mitochondrial morphology was assessed in mouse embryonic fibroblasts (MEFs), before or after transduction with retroviruses encoding the OSKM reprogramming factors for the indicated times, by immunofluorescence (IF) with anti-Tom20 antibody (Fig. 1a). We classified cells according to their mitochondrial morphology as tubular, fragmented or mixed (Supplementary Fig. 1a). Before expression of the reprogramming factors, cells largely displayed tubular mitochondrial morphology (Fig. 1a, graphic and right panels). Interestingly, mitochondrial fission ensued when cells were transduced with OSKM-encoding retroviruses, peaking at day 3.5 with 55% of the cells presenting fragmented mitochondria. Conversely, the percentage of cells with tubular mitochondrial morphology decreased to 15% at day 3.5. From this day onwards, mitochondrial fragmentation decreased steadily to ∼15% at day 8 of the process and cells slowly regained a tubular mitochondrial morphology that reached a plateau at day 7, with 50% of the cells displaying a networked mitochondrial structure. Altogether, these results indicate that OSKM expression induced mitochondrial fission early in reprogramming.

During reprogramming cells undergo mesenchymal to epithelial transition^35^,^36^. Alexa Fluor 488-Phalloidin staining of cultures from day 6 following OSKM transduction of MEFs showed scattered cells displaying thick, parallel bundles of F-actin characteristic of mesenchymal cells (Fig. 1b, leftmost panels) and isolated epithelial-like colonies displaying thin cortical bundles of F-actin (Fig. 1b, middle and right). Mesenchymal cells presented a mitochondrial morphology distribution similar to that found at day 0 (compare graph in Fig. 1b left bar to that in Fig. 1a), whereas over 80% of epithelial-like cells displayed fragmented mitochondria (Fig. 1b, right bar). Ultrastructural analysis of the cultures by transmission electron microscopy at different days of reprogramming showed a gradual reduction in mitochondrial size ranging from ∼1 μm on day 0 to 0.5 μm in cells constituting the epithelial-like colonies on day 8 of reprogramming (graph in Fig. 1c). Other than in size, we did not observe any further differences in the internal structure of the mitochondria between cells at different stages of reprogramming (Fig. 1c, left). Interestingly, epithelial-like colonies displayed a mitochondrial morphology similar to that found in pluripotent cells, where all cells presented fragmented mitochondria (Fig. 1d), indicating that a mitochondrial morphology characteristic of the pluripotent state is established during the early phases of reprogramming, before acquiring or just after achieving epithelial-like morphology. These results also suggest that mitochondrial fission early in reprogramming is critical for initiating the transit to pluripotency.

### Reprogramming intermediates contain fragmented mitochondria

**Figure 2:**
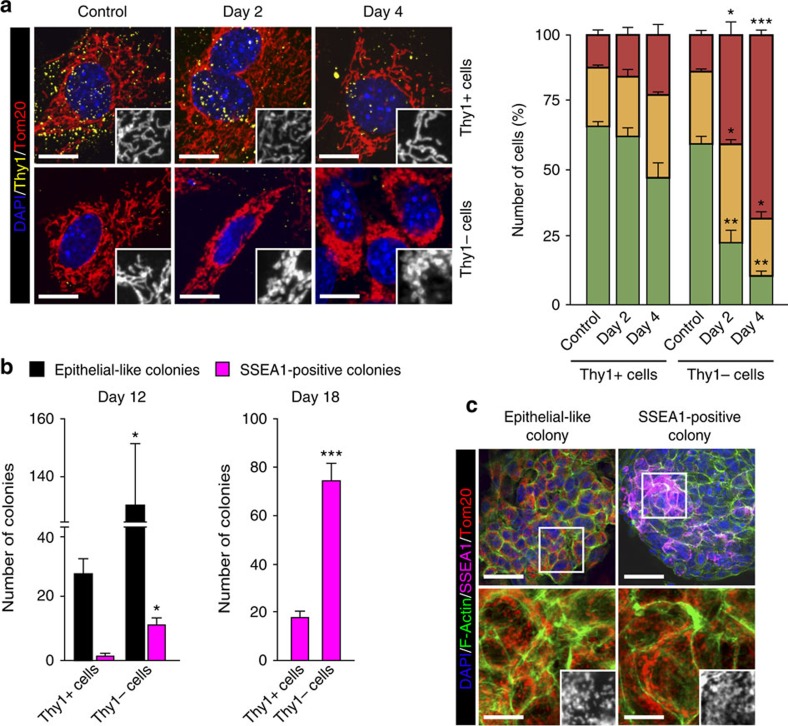
Mitochondrial fission is associated with cells undergoing reprogramming. (**a**) MEFs were mock-infected (control) or transduced with the reprogramming factors. At the indicated days, cells were fixed and Thy1 expression and mitochondrial morphology assessed by IF. (left) Representative confocal images of MEFs stained with anti-Thy1 (yellow) and anti-Tom20 (red) antibodies before (control) or after expressing the OSKM factors for the indicated days. Insets show a black and white magnification of the pictures. DAPI (blue) was used as a nuclear counterstaining. Scale bar, 16 μm. Graph on the right shows the quantification of the different mitochondrial morphologies observed in MEFs before (control) or at the indicated days after OSKM expression in the Thy1-positive and -negative cells (*n*=3). (**b**) MEFs were mock-infected (control) or transduced with the reprogramming factors. At day 4, cells were sorted as Thy1-positive and -negative. Sorted Thy1-positive and -negative cell populations were further cultured for 8 or 14 days, and epithelial-like and SSEA1-positive colonies were identified by IF analysis using Alexa Fluor 488-Phalloidin and anti-SSEA1 antibody. Graph shows the quantification of epithelial-like and SSEA1-positive colonies found at the indicated days of reprogramming (sorted at day 4) (*n*=3). (**c**) Representative confocal images of epithelial-like and SSEA1-positive colonies found in OSKM-transduced MEFs at days 12 and 18 of reprogramming stained with Alexa Fluor 488-Phalloidin (green, to stain F-actin), anti-Tom20 (red, to label mitochondria) and anti-SSEA1 (violet, to label pluripotent colonies) antibodies. (lower) Higher magnifications of the indicated area in the respective upper images. Insets in the lower images are a black and white magnification of the pictures to illustrate mitochondrial morphology in colonies. Scale bar, 24 μm (upper); scale bar, 8 μm (lower), respectively. Data are represented as mean±s.e.m. (*P<0.05, **P<0.01, ***P<0.001). One-tailed unpaired Student's *t*-test was used to compare data sets.

Somatic cells prone to undergo cell reprogramming downregulate the somatic Thy1 surface marker (Supplementary Fig. 1b,c) very early upon expression of reprogramming factors^37^,^38^. We next sought to investigate whether these first cellular intermediates of cell reprogramming underwent mitochondrial fission. For this, we first analysed mitochondrial morphology in Thy1-positive and -negative cells during the early stages of reprogramming by IF. Expression of OSKM in MEFs caused a small change in the proportion of Thy1-positive cells that displayed fissioned mitochondria during the first days of reprogramming. Remarkably, the percentage of Thy1-negative cells displaying fragmented mitochondria greatly increased during the first 4 days reprogramming (Fig. 2a). These results suggest that mitochondrial fragmentation is associated with cells prone to undergo full cell reprogramming. To test this further, we sorted cells at day 4 of reprogramming based on Thy1 expression and followed the Thy1-positive and -negative cell populations along the cell reprogramming path. Strikingly and in contrast to the Thy1-positive population, we observed that the Thy1-negative cell population gave rise to about 4-fold more epithelial-like and SSEA1-positive colonies than the Thy1-positive counterparts at days 12 and 18 of reprogramming, respectively (Fig. 2b). Cells in both epithelial-like and SSEA1-positive colonies displayed fragmented mitochondria (Fig. 2c). Altogether, these results support the hypothesis that mitochondrial fission is intimately linked to successful cell reprogramming to pluripotency.

Although we detected a minor decrease in mitochondrial mass by flow cytometry or Tom40 immunoblotting, we neither observed an induction of LC3B-dependent autophagy, nor mitophagy early in cell reprogramming (Supplementary Figs 1d–f and 2a–c). In fact, we detected an inhibition of LC3B-mediated autophagy during the early stages of the cell reprogramming process in a time-dependent manner, as measured by the amount of recombinant GFP-LC3B puncta and processing of endogenous LC3B in the cultures (Supplementary Fig. 1d,e). Genes involved in mitochondrial biogenesis showed increased expression at later stages of reprogramming (Supplementary Fig. 2d). Altogether, these results indicate that OSKM-induced mitochondrial fission was not associated with mitophagy.

### OSKM-induced mitochondrial fission is Drp1-dependent

**Figure 3:**
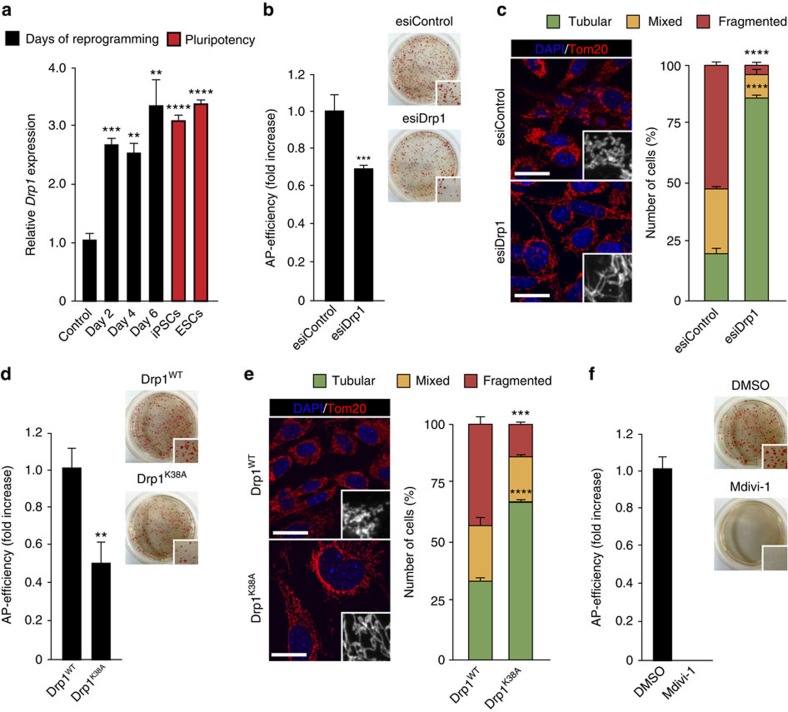
Reprogramming-induced mitochondrial fission follows a Drp1-dependent pathway. (**a**) Total RNA was extracted from wild-type MEFs left untreated (control) or OSKM-infected for the indicated days (black bars), or from the indicated pluripotent cells (red bars). The expression of *Drp1* gene was then assessed by qPCR and represented as relative gene expression normalized to control MEFs (*n*=3). (**b**,**d**,**f**) Graphs showing the number of AP-positive colonies obtained after 25 days of retroviral delivery of the OSKM factors in the presence of (**b**) esiRNA control (esiControl) or esiRNA targeting *Drp1* (esiDrp1), (**d**) Drp1 wild-type (Drp1^WT^) or the catalytically inactive K38A mutation (Drp1^K38A^) or (**f**) the Drp1 inhibitor Mdivi-1 (50 μM) (*n*=6). (right) Representative bright-field images from the plates of the indicated cultures after AP-staining. Insets show a magnification of a selected area from the AP-stained plates. (**c**,**e**) Representative confocal images of MEFs expressing the reprogramming factors during 4 days in the presence of the indicated (**c**) esiRNAs or (**e**) Drp1 constructs and stained with anti-Tom20 antibody (red) to assess the indicated mitochondrial morphologies. Insets show a black and white magnification of the pictures. DAPI (blue) was used as a nuclear counterstaining. Scale bars, 24 μm. Graphs on the right of the images show the quantification of the indicated mitochondrial morphologies observed in cells treated as above (*n*=3) Data are represented as mean±s.e.m. (**P<0.01, ***P<0.001, ****P<0.0001). One-tailed unpaired Student's *t*-test was used to compare data sets. qPCR, quantitative PCR.

To gain insight into the gene network controlling mitochondrial dynamics during reprogramming, we examined the expression of factors, known to play a role in the regulation of this process^18^, in MEFs, OSKM-expressing cells and pluripotent cells. Relative to their expression at day 0 in MEFs, the expression of the pro-fission factors *Gdap1*, *Fis1*, *Mff*, *MiD49* and *MiD51* (Supplementary Fig. 3a), with the exception of *Drp1* that increased threefold (Fig. 3a), was lower in pluripotent cells. In the assessment of pro-fusion factors we found that, compared with day 0, the mRNA levels of *Opa1* did not change in pluripotent cells (Supplementary Fig. 3a and see below), while the expression of *Mfn1* and *Mfn2* was either comparable to the levels found in control MEFs (*Mfn1*) or decreased by more than 75% (*Mfn2*) in pluripotent cells (Supplementary Fig. 3a and see below). Interestingly, the expression of the majority of the factors involved in mitochondrial dynamics increased during reprogramming (Fig. 3a and Supplementary Fig. 3a), reflecting the early adjustment of mitochondrial shape and function induced upon expression of reprogramming factors.

As Drp1 was the only pro-fission factor showing an increase in its expression during reprogramming and in pluripotent cells, we hypothesized that OSKM-induced mitochondrial fission could follow a pathway dependent on the activity of this dynamin-related protein. To address the importance of Drp1 in this process, we carried out reprogramming assays in which Drp1 function was targeted by three different experimental approaches: RNA interference (Fig. 3b,c), overexpression of a dominant negative Drp1 mutation (Fig. 3d,e) and chemical inhibition of Drp1 GTPase enzymatic activity (Fig. 3f and Supplementary Fig. 3c). We then evaluated reprogramming efficiency by scoring colonies positive for Alkaline Phosphatase (AP) 25 days after OSKM expression. Inhibition of Drp1 function by either reduction of *Drp1* mRNA and protein levels by endoribonuclease-prepared small interference RNAs (esiRNAs) targeting Drp1 (Supplementary Fig. 3b), overexpression of the Drp1^K38A^ dominant negative mutation or treatment of the cells with the Drp1 inhibitor Mdivi-1 reduced the appearance of AP-positive colonies by 35% (Fig. 3b), 50% (Fig. 3d) or 100% (Fig. 3f), respectively, compared with their corresponding controls. Furthermore, targeting the function of this dynamin-related protein as described above impaired OSKM-induced mitochondrial fission (Fig. 3c,e and Supplementary Fig. 3c). Altogether, these results suggest that reprogramming-induced mitochondrial fission follows a Drp1-dependent pathway.

### OSKM expression induces Drp1-S579 phosphorylation

**Figure 4:**
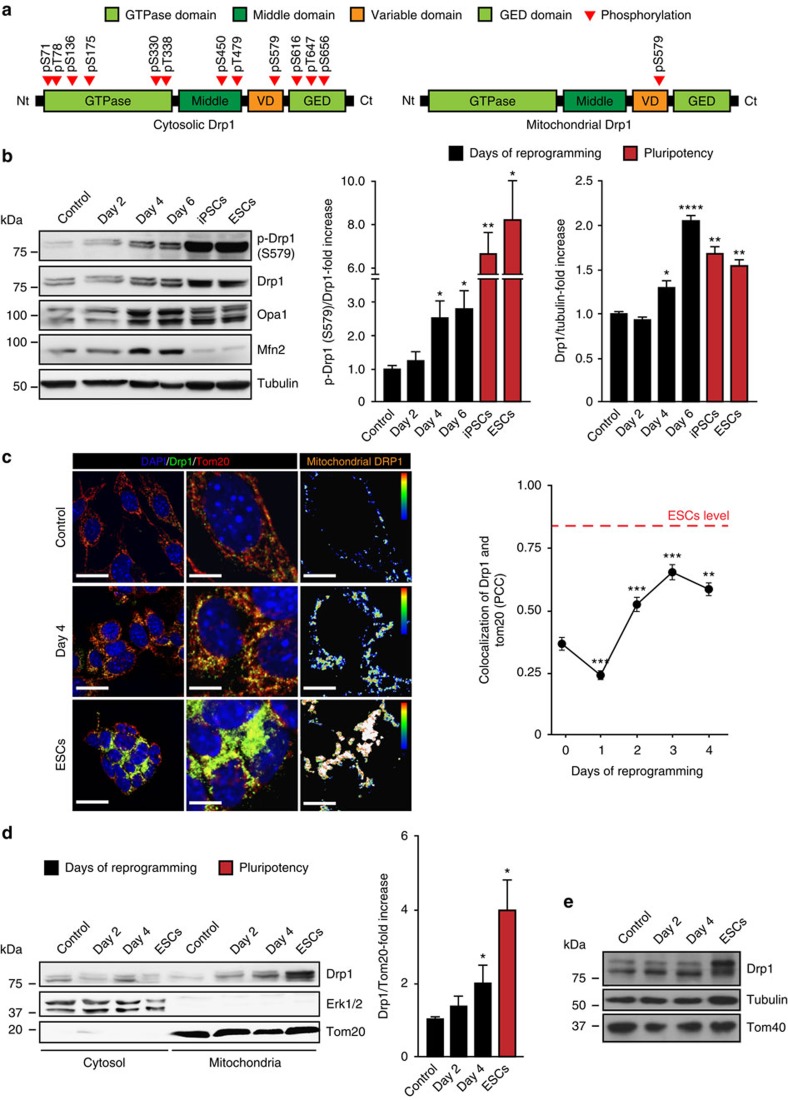
OSKM-induced Drp1 phosphorylation at serine 579. (**a**) Diagram depicting the identified phosphorylated residues in cytosolic- (left) or mitochondria- (right) associated Drp1 in self-renewing ES cells by LC-MS/MS. (**b**) Lysates of mock- or OSKM-transduced MEFs for the indicated days, or the specified pluripotent cells were analysed by immunoblotting using the indicated antibodies. Graphs on the right show the quantification of the indicated ratios (*n*=3). (**c**) Representative confocal images of MEFs before (control, upper) or 4 days after OSKM expression (day 4, middle), or ES cells (ESCs, lower) stained with anti-Drp1 (green) or anti-Tom20 (red) antibodies. DAPI (blue) was used as a nuclear counterstaining. (middle) Magnification of the pictures on the left. Rightmost pictures are colour map representations of the pictures in the middle panels to display colocalized pixels between both fluorophores according to the displayed colour bar. Warm colours depict pixels with highly correlated intensity and spatial overlap while cold colours are indicative of random or anti-correlation. Scale bars, 24 μm (left); scale bar, 12 μm (middle); and scale bar, 12 μm (right). Graph on the right shows the quantification of the PCC to display the degree of colocalization between Drp1 and Tom20 at the indicated days of reprogramming. Red dashed line indicates the levels of Drp1 and Tom20 colocalization found in ES cells (*n*=3). (**d**) Cells treated as in **b** were lysed at the indicated days and fractionated into cytosolic or mitochondrial subcellular fractions. Then, subcellular fractions were subjected to immunoblotting analysis using the indicated antibodies. Graph shows the quantification of the Drp1/Tom20 co-fractionation ratio in the mitochondrial fraction (*n*=3). (**e**) Immunoblot showing the amount of the indicated proteins in total lysates from cells used for subcellular fractionation as control. Data are represented as mean±s.e.m. (*P<0.05, **P<0.01, ***P<0.001, ****P<0.0001). One-tailed unpaired Student's *t*-test was used to compare data sets. LC-MS/MS, liquid chromatography with tandem mass spectrometry.

Analysis by liquid chromatography with tandem mass spectrometry (LC-MS/MS) of mitochondria-associated and cytosolic Drp1 in ES cells showed that phospho-Drp1-S579 is the only phosphorylated form of Drp1 associated with these organelles, whereas cytosolic Drp1 showed a wide variety of phosphorylated residues (Fig. 4a and Supplementary Fig. 4a). Indeed, many of the phosphorylation modifications found in cytosolic Drp1 have not been previously described and warrant further investigation into their regulatory role and cell-type specificity. We then examined whether the phosphorylation of serine 579 in mouse Drp1 (Drp1-S579), known to favour its recruitment to mitochondria in response to proliferative signals^19^, played a role in reprogramming-induced mitochondrial fragmentation. Remarkably, during reprogramming and compared with mock-transduced MEFs, immunoblotting analysis showed that Drp1-S579 phosphorylation increased both in a time-dependent manner upon OSKM expression and in pluripotent cells (Fig. 4b and Supplementary Fig. 4b). In agreement with the mRNA expression data showed above, Drp1 and Opa1 proteins also increased during reprogramming and in pluripotent cells, whereas Mfn2 protein levels increased during reprogramming and markedly decreased in pluripotent cells (Fig. 4b and Supplementary Fig. 4b). Overall, our findings suggest that mitochondrial fission triggered by the reprogramming factors uses a similar set of proteins to that found in somatic cells and that the dynamics of these organelles in pluripotent cells could rely on high levels of active Drp1 and low expression of Mfn2.

Strikingly, IF and subcellular fractionation analysis showed that Drp1 increased the colocalization with mitochondrial markers during cell reprogramming, compared with proliferating MEFs (Fig. 4c–e). Interestingly, Drp1 showed a marked colocalization with mitochondria in self-renewing ES cells (Fig. 4c, graph on the right). These results overall suggest that OSKM expression stimulates the recruitment of Drp1 to mitochondria during reprogramming by inducing the phosphorylation of Drp1-S579.

### OSKM-induced mitochondrial fission depends on ERK signalling

**Figure 5:**
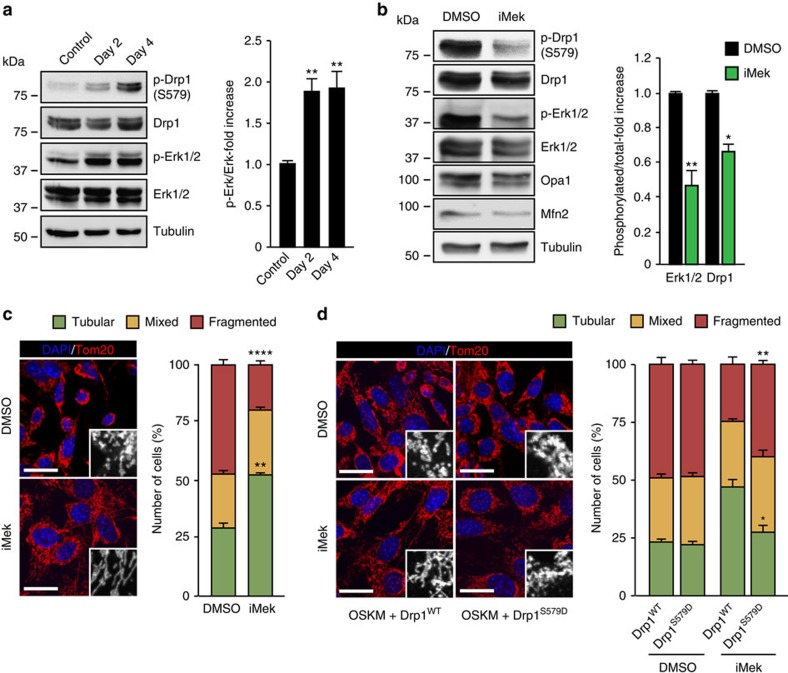
Reprogramming-induced mitochondrial fission depends on Erk1/2 phosphorylation of Drp1. (**a**) Lysates of mock- or OSKM-transduced MEFs for the indicated days were analysed by immunoblotting using the indicated antibodies. Graphs on the right show the quantification of the indicated ratios (*n*=3). (**b**) MEFs were OSKM-transduced and 3 days post-infection cells were treated with DMSO (black bars), as vehicle control, or the MEK1/2 inhibitor PD0325901 (1 μM) (iMek, green bars) for 16 h. Then, cell lysates were prepared and analysed by immunoblotting using the indicated antibodies (left). Graphs on the right show the quantification of the indicated ratios (*n*=3). (**c**) (left) Representative confocal images of OSKM-expressing MEFs for 3 days, treated as in **b** and stained with anti-Tom20 antibody (red) to assess the different mitochondrial morphologies. Insets show a black and white magnification of the pictures. DAPI (blue) was used as a nuclear counterstaining. Scale bars, 24 μm. Graph on the right shows the quantification of the indicated mitochondrial morphologies observed in the cells treated as indicated (*n*=3). (**d**) (left) Representative confocal images of MEFs expressing the reprogramming factors, together with Drp1 wild type (Drp1^WT^) or the phosphomimetic S579D mutation (Drp1^S579D^), during 4 days. Cells were then treated, fixed and stained as in **c**. Insets show a black and white magnification of the pictures. DAPI (blue) was used as a nuclear counterstaining. Scale bars, 24 μm. Graph on the right shows the quantification of the indicated mitochondrial morphologies observed in the cells treated as indicated (*n*=3). Data are represented as mean±s.e.m. (*P<0.05, **P<0.01, ****P<0.0001). One-tailed unpaired Student's *t*-test was used to compare data sets.

It has recently been shown that ERK2 phosphorylated DRP1-S616 in human cells (the equivalent to S579 in mouse Drp1) (refs 28, 29). We next investigated the possibility that Erk1/2-mediated Drp1-S579 phosphorylation could play a role in reprogramming-induced mitochondrial fission. Indeed, immunoblotting analysis showed that Erk1/2 phosphorylation during the first 4 days of reprogramming paralleled that of phospho-S579-Drp1 (Fig. 5a). In addition, 16 h incubation of OSKM-transduced cells with the specific MEK1/2 inhibitor PD0325901 decreased Erk1/2 activation by 50% and phospho-Drp1-S579/total Drp1 protein ratio by 35% relative to mock-treated controls (Fig. 5b). Moreover, IF analysis showed that inhibition of Erk1/2 activity by the MEK1/2 inhibitor decreased mitochondrial fragmentation compared with mock-treated control (Fig. 5c). Treatment with the MEK inhibitor did not affect Opa1 or Mfn2 protein levels (Fig. 5b), suggesting that the inhibition of OSKM-induced mitochondrial fission was due to the decrease in the Erk1/2-mediated Drp1-S579 phosphorylation. Supporting this, expression of Drp1 containing the phosphomimetic S579D mutation (Drp1^S579D^) rescued OSKM-induced mitochondrial fragmentation in cells incubated with the MEK inhibitor, whereas expression of the Drp1^WT^ did not (Fig. 5d). Although a contribution of Cdk1 to the phosphorylation of Drp1-S579 cannot be completely ruled out^19^, our results indicate that the activation of the MAP-kinases Erk1/2 by the reprogramming factors is required for OSKM-induced mitochondrial fission.

### OSKM expression activates Erk1/2 by reducing Dusp6 levels

**Figure 6:**
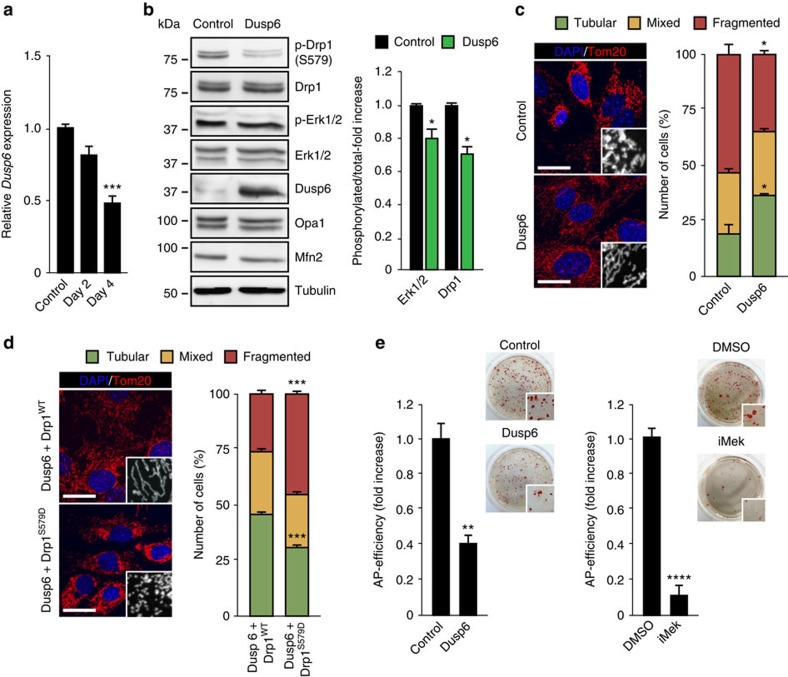
Effect of Dusp6 in cell reprogramming. (**a**) *Dusp6* gene expression in untreated (control) or OSKM-infected MEFs was assessed by qPCR (*n*=3). (**b**) MEFs were transduced with OSKM together with empty vector- or Dusp6-encoding retroviruses. Four days after, cell lysates were prepared and analysed by immunoblotting using the indicated antibodies. Graphs on the right show the quantification of the data (*n*=3). (**c**) (left) IF images of MEFs transduced as in **b** and stained with anti-Tom20 antibody (red) 4 days post infection to assess the different mitochondrial morphologies. Insets show a black and white magnification of the pictures. DAPI (blue) was used as a nuclear counterstaining. Scale bars, 24 μm. Graph on the right shows the quantification of the observed mitochondrial morphologies in the cells (*n*=3). (**d**) (left) IF images of MEFs co-expressing the reprogramming factors and Dusp6 together with Drp1 wild type (Drp1^WT^) or the phosphomimetic S579D mutation (Drp1^S579D^) during 4 days. Cells were then fixed and stained as in **c**. Insets show a black and white magnification of the pictures. DAPI (blue) was used as a nuclear counterstaining. Scale bars, 24 μm. Graph on the right, quantification of the indicated mitochondrial morphologies observed in the cells (*n*=3). (**e**) Graphs showing the number of AP-positive colonies obtained in MEFs after 25 days of retroviral delivery of the OSKM factors either together with empty vector- (control) or Dusp6-encoding retroviruses (Dusp6) (left) or in the presence of DMSO (as vehicle control) or the MEK1/2 inhibitor PD0325901 (1 μM) (iMek) (right) (*n*=3). Panels in the right, bright-field images from the plates of the indicated cultures after AP-staining. Insets show a magnification of a selected area from the AP-stained plates. Data are represented as mean±s.e.m. (*P<0.05, **P<0.01, ***P<0.001, ****P<0.0001). One-tailed unpaired Student's *t*-test was used to compare data sets. qPCR, quantitative PCR.

**Figure 7:**
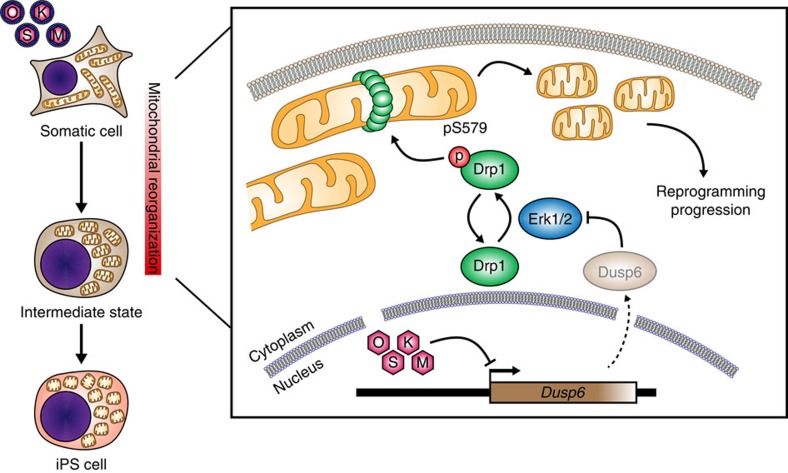
Model. The illustration depicts the role of ERK signalling in activating Drp1 through its phosphorylation at S579 during early reprogramming. The role of *Dusp6* downregulation by reprogramming factors in activating ERK signalling during early reprogramming is also displayed.

Members of the DUSP family of protein phosphatases control intracellular MAP-kinase activity, and the expression level of DUSP phosphatases is inversely proportional to levels of MAP-kinase activity^30^,^31^. Interestingly, we observed that the mRNA levels of one the major Erk cytosolic phosphatases, *Dusp6*, decreased early during reprogramming in a time-dependent manner (Fig. 6a). This suggests that the observed decrease in *Dusp6* expression could lead to ERK signalling activation early in reprogramming. In fact, expression of recombinant *Dusp6* decreased both Erk1/2 activation and Drp1-S579 phosphorylation induced by OSKM expression (Fig. 6b). In line with these findings, Dusp6 overexpression decreased OSKM-induced mitochondrial fission by about 50% compared with the control cells (Fig. 6c), without affecting Opa1 or Mfn2 protein levels (Fig. 6b). Remarkably, expression of Drp1^S579D^ together with OSKM rescued reprogramming-induced mitochondrial fission, whereas Drp1^WT^ did not (Fig. 6d). Furthermore and relative to control MEFs co-transduced with empty vector and OSKM-encoding retroviruses, the addition of *Dusp6* to the reprogramming cocktail reduced the appearance of AP-positive colonies by 60% (Fig. 6e, graph on the left). A similar effect was observed when reprogramming was carried out in the presence of a MEK1/2-inhibitor (Fig. 6e, graph on the right). Taken together, these results propose a role for *Dusp6* downregulation in the early activation of Erk1/2 to drive the Drp1-dependent mitochondrial fragmentation necessary for cell reprogramming (Fig. 7).

## Discussion

During cell reprogramming, somatic cells undergo a profound reorganization in the number and shape of mitochondria that reflects the transition from a somatic oxidative- into a pluripotent glycolytic-based metabolism^39^. In this study, we uncovered a mitochondrial fission process, orchestrated by ERK signalling to activate Drp1, that constitutes an early and necessary step for efficient cell reprogramming to pluripotency.

The bioenergetics switch observed during reprogramming takes place during the mid-to-late stages of the process^32^ and our data indicate that reprogramming-induced mitochondrial fission precedes this metabolic change. Notably, mitochondrial fission promotes a shift to aerobic glycolysis in different cells types, including neuroblastoma^40^, vascular smooth muscle cells^41^ and cancer-associated myofibroblasts^42^. Furthermore, a number of studies have proposed a pro-tumourigenic role for mitochondrial fission^28^,^29^,^40^,^43^,^44^,^45^,^46^,^47^,^48^,^49^. Thus, it is possible that reprogramming-induced mitochondrial fission could be one of the first events promoting a metabolic rewiring during this process, favouring the transition towards a glycolytic-based metabolism necessary to support the rapid proliferation of pluripotent cells.

Compared with their somatic counterparts, iPS cells show a reduction in the number and complexity of mitochondria. Although mitochondrial fission is part of the quality-control mechanism whereby damaged mitochondria are eliminated by mitophagy^18^, our results indicate that this specific form of autophagy was not activated during the early steps of reprogramming. In contrast, transient activation of autophagy was shown to reduce mitochondrial number very early in reprogramming^50^. It is possible that the activation peak of this degradative process observed by Wang *et al*. went unnoticed in our experimental settings due to its rapid and transient nature. On another hand and in agreement with our results, it has recently been shown that LC3/Atg5-dependent autophagy is not responsible for mitochondrial remodelling during reprogramming^51^,^52^. These observations altogether suggest that the reduction in the quantity and complexity of mitochondria occurs at later during reprogramming, possibly through an adaptive process to the growth conditions required for maintaining self-renewal of highly proliferative pluripotent cells and/or through an Atg5-independent autophagy pathway^52^. Surprisingly, Drp1 was recently reported to be dispensable for cell reprogramming^53^. Conversely and in agreement with our results, inhibition of Drp1 function by either pharmacological inhibition or short-hairpin RNA-mediated knockdown impaired reprogramming in human cells^54^,^55^. These paradoxical results could be explained by the incomplete knockdown of Drp1 achieved by Wang *et al*.^53^ in their experiments.

Inhibition of ERK signalling has been a key finding to define a unique ground state of pluripotency in ES cells, and the supplementation of culture media with MEK1/2 inhibitors to impair ERK signalling has been instrumental in the derivation of ES cells from certain mouse strains or other species^56^,^57^,^58^. Accordingly, inhibition of ERK signalling during late stages of reprogramming favoured the acquisition of pluripotency, while early inhibition of these kinases impaired the process of cell reprogramming^59^. Thus, ERK signalling plays opposing roles at different phases of cell reprogramming. ERK signalling favours ES cell differentiation by destabilizing the pluripotency network and targeting poised chromatin to developmental genes and, by doing so, sensitizing pluripotent cells to instructive differentiation signals^60^,^61^. Therefore, this effect may explain the positive effect of ERK inhibition in stabilizing the pluripotent state throughout the late stages of reprogramming. On the other hand, and given the importance of ERK signalling in maintaining cellular homeostasis, these kinases may play a key role during the initial phase of cell reprogramming by orchestrating a coordinated response in somatic cells to undertake the dramatic changes elicited by reprogramming factors, before the pluripotent network is induced. In this regard, activation of ERK signalling by constitutive expression of a constitutively active K-Ras mutation confers on cells a large degree of phenotypic plasticity that promotes their neoplastic transformation and acquisition of stem cell characteristics^62^. Thus, it is likely that ERK signalling could play significant roles during the initial stages of cell reprogramming, which may pave the way for initiating the transit to the pluripotent state. We propose that one of these critical functions carried out by ERK signalling in this early phase of OSKM-induced cell reprogramming is promoting Drp1-dependent mitochondrial fission. Interestingly, OSKM-dependent repression of several members of the DUSP family very early in cell reprogramming has been shown^63^,^64^. Thus and although the participation of alternative mechanisms cannot be completely ruled out, we propose a role for *Dusp6* downregulation in the activation of ERK signalling early in OSKM-induced cell reprogramming. In contrast, induction of *Dusp2* and *Dusp7* gene expression controls ERK signalling activation and pluripotency in mouse ES cells^65^. And knockdown of Dusp7 impairs mouse ES cell self-renewal^66^. Therefore, transcriptional modulation of different DUSP family members is likely to act as a common mechanism for regulating ERK signalling, which plays opposing roles in somatic or pluripotent cellular states. On another hand, DRP1 phosphorylation by ERK has been shown to play an important role in cellular transformation elicited by oncogenic RAS or BRAF mutations in pancreatic cancer^28^,^29^. Interestingly, activation of ERKs in pancreatic cancer and cell reprogramming takes place at different levels of the pathway: upstream by RAS or BRAF mutations and at the level of MAP-kinases by Dusp6 downregulation, respectively. Thus, although DRP1 activation by ERK signalling is necessary for both processes, additional cascades activated by the upstream ERK activators, which may play a role in cellular transformation, may not be involved in the process of cell reprogramming.

Although iPS cells can be obtained from any cell type, cell reprogramming is a very inefficient process^3^. Both deterministic and stochastic models for cell reprogramming have been proposed^67^. However, the distinctive outcome upon expression of reprogramming factors may reflect the differential cellular responses to the forced expression of these factors, which could largely depend on particular physiological conditions, including cellular organization, cell cycle phase or metabolic dependence. In this regard, cells undergoing reprogramming constitute a privileged subset of cells with an ultrafast cell cycle^68^. Research on the cytoarchitecture, gene expression and metabolic profiles of rapidly cycling cells could both shed light into the advantages of these cells over the normal cell population and help to understand the processes that normal cycling cells must undertake to initiate their transit to pluripotency upon expression of reprogramming factors. Finally, as cell reprogramming and tumourigenesis are gradually showing many parallels^69^,^70^, our findings may also be relevant for understanding the initial events leading to human malignancies^71^.

## Methods

### Cell culture, reprogramming assays, reagents and plasmids

E14Tg2a and CCE1.19 ES cells were cultured on gelatinized plates in ES cell medium supplemented with 10% FBS (Hyclone) in the presence of LIF^72^. When indicated, ES and iPSCs were grown on gelatinized plates in 2i medium^57^ ((1:1) mixture of Neurobasal:DMEMF12 (both from Life Technologies) supplemented with 0.5 × N2, 0.5 × B27 (both from Life Technologies), 3 μM CHIR99021 and 1 μM PD0325901 (both from Calbiochem)) in the presence of LIF. PlatE and SNL cells^1^ were grown in DMEM containing 10% FBS. When indicated, SNL cells were mitotically inactivated by treatment with 10 μg ml^−1^ Mitomycin-C (Sigma-Aldrich) for 2.5 h at 37 °C. Wild-type MEFs (homogenous C57BL/6 background) were prepared from E13.5 pooled embryos and cultured in DMEM supplemented with 10% FBS and penicillin/streptomycin. Chloroquine (Sigma-Aldrich) was used as indicated. All cells have been routinely tested for mycoplasma contamination using the Lookout Mycoplasma PCR detection kit (Sigma-Aldrich). The retroviral vectors pMX-Oct4, pMX-Sox2, pMX-Klf4 and pMX-c-Myc^1^, pcDNA3.1(+) encoding mouse *Drp1* wild type from Dr David Chan's laboratory and pMXs-IP-EGFP-LC3B (ref. 73) were from Addgene. Flag-tagged *Drp1* was subcloned into pPYCAG-IP by PCR following standard procedures. *Drp1*^*K38A*^ and *Drp1*^*S579D*^ cDNA was obtained by site-directed mutagenesis using PCR, followed by DNA sequencing. The cDNAs encoding rat *Dusp6* was a gift from Dr Rafael Pulido. *Drp1*^*WT*^, *Drp1*^*K38A*^, *Drp1*^*S579D*^ and *Dusp6* cDNAs were subcloned into pMXiE retroviral vector by PCR using standard procedures. esiRNAs targeting GFP (as negative control) or Drp1 were from Sigma-Aldrich.

Reprogramming was carried out by transduction of MEFs with retroviruses encoding Oct4, Sox2, Klf4 and c-Myc as previously described^1^. Ecotropic retroviruses were produced in PlatE cells transfected using Polyethylenimine (PEI) ‘Max' (Mw 40,000) (Polysciences) exactly as described^1^. For reprogramming, 8 × 10^5^ MEFs were plated per p100 mm the day before the assay. Next day (day 0), MEFs were incubated overnight with a 1:1:1:1 mixture of mouse Oct4, Sox2, Klf4 and c-Myc retroviral supernatants supplemented with 4 μg ml^−1^ Polybrene. Next day, the supernatants were replaced with fresh media and cells were incubated for 3 more days (day 4). Then, 5 × 10^4^ cells were plated on a confluent layer of mitotically inactivated SNL feeders seeded the day before on gelatine-coated p60 mm at 2.5 × 10^6^ cells per dish. Next day (day 5), media was changed to ES cell growth media containing 15% FBS and LIF. Media was changed every other day. When indicated cell reprogramming was conducted in the presence of DMSO (as control), 1 μM of the MEK1/2 inhibitor PD0325901 or 50 μM of the Drp1 inhibitor Mdivi1 (Millipore). For reprogramming in the presence of esiRNAs, 3 × 10^4^ MEFs in p24 multi-well plates were transduced as before with OSKM. Infected MEFs were then transfected overnight with 0.6 μg of the indicated esiRNAs at days 1 and 3 post infection, using lipofectamine RNAiMAX (Life Technologies). Reprogramming was assessed 25 days after transduction of MEFs with OSKM-encoding retroviruses by scoring all the alkaline phosphatase positive colonies per p60 mm. Alkaline phosphatase staining was performed according to the manufacturer's instructions (Alkaline Phosphatase Detection Kit, SCR004, Millipore).

For iPSC generation, colonies were handpicked at day 25 of reprogramming, transferred to a 1.5 ml tube containing 50 μl of Trypsin-EDTA solution and incubated for 10 min at 37 °C. Then, cells were disaggregated with a pipette by adding 200 μl of ES cell media containing 10% FBS and LIF. Cells were then plated on SNL feeders in p24 multi-well plates (1 clone per well) in ES cell media supplemented with 10% FBS and LIF. When colonies were macroscopically visible, the media was changed to 2i medium with LIF. Surviving clones were further expanded in 2i media with LIF and analysed as indicated. More than 80% of the pluripotent cells per clone were shown to contain 20 chromosomes. Chromosome counting was carried out exactly as described^74^ using DAPI for staining DNA. At least 20 spreads per sample were analysed. Pluripotent cells that passed all the quality tests (Supplementary Fig. 5) were selected and used as described in the text.

### Immunofluorescence and flow cytometry

For IF analysis all cells were plated on gelatine-coated coverslips. Cells, untreated or transduced with the indicated viruses, were plated at 1.5 × 10^4^ cells cm^−2^ the day before processing. Then, cells were fixed for 20 min at room temperature with 4% paraformaldehyde in PBS, permeabilized for 10 min with 0.5% Triton X-100 in PBS, blocked for 30 min with blocking buffer (3% Bovine Serum Albumin, BSA, in PBS containing 0.025% Tween-20) and incubated overnight with primary antibodies in blocking buffer. After washing with PBS supplemented with 0.025% Tween-20, cells were incubated for 1 h with the appropriate secondary antibodies in blocking buffer containing 2 μg ml^−1^ of DAPI (Invitrogen), washed overnight with PBS, mounted with MOWIOL and analysed using confocal microscopy. Confocal IF images were taken using a Fluoview FV10i confocal microscope equipped with 405-, 488- and 633-nm lasers (Olympus). Three-dimensional reconstructions of *z*-stacks and colour map representations of the images were performed using FV10-ASW 2.1 viewer software. All images were compiled using Adobe Illustrator CS5.

Colocalization of Tom20 and GFP-LC3B or Tom20 and Drp1 stainings was evaluated by calculating the Pearson Correlation Coefficient (PCC) using the freely available JACoP plug-in ((http://rsb.info.nih.gov/ij/plugins/track/jacop.html) for ImageJ analysis software, as previously described^75^.

For detection of Filamentous (F)-Actin cells were incubated with Alexa Fluor 488- or Alexa Fluor 555-conjugated Phalloidin (# A12379 or #A22283, respectively; Life technologies; used at 1:50 dilutions).

For mitochondrial mass assessment by flow cytometry, cells were trypsinized and resuspended in media containing 25 μg ml^−1^ of the membrane potential-independent mitochondrial dye MitoTracker Green FM (M7514, Life technologies), incubated at room temperature for 10 min and then processed for flow cytometry. All measurements were taken using a BD FACSCanto II or FACS Verse flow cytometers (both from BD Biosciences) and analysed using FlowJo software (Tree Star Inc.). At least 10,000 events from each sample were recorded. Cell sorting was performed exactly as described^72^, using the indicated antibodies. Briefly, cells were trypsinized, filtered through a 30 μm cell strainer and counted. Cells were spun down at 250 g for 5 min and resuspended at 10–20 × 10^6^ cells per ml in blocking buffer (2% FCS in PBS). A total of 100 μl of the cell suspension (1–2 × 10^6^ cells) was added to round-bottom 5 ml tubes and incubated for 15 min on ice. Then, 100 μl of a 2 × solution containing the primary antibodies in blocking buffer was added to the tubes and incubated on ice for 45 min. Tubes were filled with ice-cold PBS, contents mixed by inverting the tubes and cells spun down at 250 g for 5 min. Cell pellets were then resuspended in 200 μl of a solution containing the secondary antibodies diluted in blocking buffer and incubated for 30 min on ice. Cells were spun down as before, resuspended in 300 μl of blocking buffer containing 1% FBS and kept on ice. Just before sorting, propidium iodide was added to the tubes at 1 μg ml^−1^ final concentration to exclude dead cells. Cell sorting was carried out using a FACS Aria cell sorter (Becton Dickinson) equipped with 407, 488 and 633 nm lasers. Details about the antibodies used in this study for IF and Flow cytometry are provided in Supplementary Tables 1 and 2.

### Transmission electron microscopy

Cell monolayers were fixed for 30 min at 4 °C in 0.1 M phosphate buffer pH 7.4, containing 2% glutaraldehyde, followed by their postfixation with 1% osmium-1% potassium ferrocyanide mixture in water for 30 min. Samples were then dehydrated with ethanol and propylene oxide and embedded in TAAB resin (T002, Taab Laboratories). Resin blocks were sectioned at 2 μm and stained with toluidine blue to identify mesenchymal cells and epithelial-like colonies in the samples. Selected semi-thin sections were then re-embedded and trimmed for ultra-thin sectioning. Ultra-thin sections were stained with lead citrate and examined in a Jeol JEM-1010 electron microscope. All images were further processed using Adobe Photoshop CS6 and compiled using Adobe Illustrator CS5.

### Imunoblotting, cell fractionation and immunoprecipitation

Cells were lysed in RIPA buffer (50 mM Tris pH 7.5, 150 mM NaCl, 0.1% SDS, 1% Triton X-100, 0.5% sodium deoxycholate, 100 mM NaF, 2 mM Na_3_VO_4_, 20 mM Na_4_P_2_0_7_ and 1 × complete proteinase inhibitor cocktail from Roche). Cellular lysates were used for immunoblotting with the indicated antibodies using standard procedures. Signals in western blots were detected using ECL prime (Amersham) and images automatically captured in an ImageQuant LAS 4000 digital imaging system equipped with FUJINON F0.85 lenses and a Fujifilm super CCD area type chip (GE). Acquired images were processed using Adobe Photoshop CS6 and analysed with ImageJ software. Details of the antibodies used in this study for WB are provided in Supplementary Tables 1 and 2. Full scans of the blots are shown in Supplementary Fig. 6.

For cell fractionation, MEFs, E14Tg2a or CCE1.19 ES cells stably expressing Flag-Drp1 or GFP (as control) were washed with ice-cold PBS, detached with a cell scraper and resuspended in hypotonic buffer (10 mM Tris-HCl pH 7.4, 0.3 M sacarose, 1 mM EDTA and 5 mM DTT) supplemented with protease (Roche) and phosphatase inhibitors (20 mM Na_4_P_2_O_7_ and 2 mM Na_3_VO_4_). Samples were then homogenized on ice using a glass Dounce pestle (three times, 30 strokes). Cellular suspension was centrifuged twice at 1,000*g*, 4 °C during 10 min to eliminate nuclei and large cellular ghosts. Supernatant was centrifuged at 13,000*g*, 4 °C for 10 min to precipitate mitochondria, supernatants were considered as the cytoplasmic fractions. Pellets containing mitochondria were subjected to two rounds of washing by resuspending the pellet in 1 ml of ice-cold hypotonic buffer followed by centrifugation at 13,000*g*, 4 °C for 10 min to precipitate mitochondria. Pellets were then resuspended in RIPA buffer as above. Cytoplasmic and mitochondrial fractions were subjected to either immunoblot analysis or immunoprecipitation with anti-Flag antibody covalently bound to magnetic beads (Sigma-Aldrich, M8823, 50 μl of slurry per ml of lysate) during 4 h at 4 °C. Beads were washed four times with ice-cold RIPA buffer followed by boiling in 1 × Laemmli sample buffer for 5 min to dissociate the antigens from the beads. Samples were then separated in 10% SDS–polyacrylamide gel electrophoresis gels. After electrophoresis, gels were stained with QC Colloidal Coomassie (Bio-Rad) and bands containing Flag-Drp1, identified based on both their migration in the gels and comparison with control wells ran in parallel, were excised, subjected to digestion with trypsin and stored at −80 °C until their analysis by mass spectrometry as indicated underneath.

### LC-MS/MS

The proteomic analysis was performed in the proteomics facility of SCSIE University of Valencia, which belongs to ProteoRed, PRB2-ISCIII, and is supported by grant PT13/0001. For LC-MS/MS, 5 μl of each sample were loaded onto a trap column (NanoLC Column, 3 μ C18-CL, 350 μm × 0.5 mm; Eksigen) and desalted with 0.1% TFA at 3 μl min^−1^ during 5 min. The peptides were then loaded onto an analytical column (LC Column, 3 μ C18-CL, 75 μm × 12 cm, Nikkyo) equilibrated in 5% acetonitrile 0.1% formic acid (FA). Peptide elution was carried out with a linear 5–35% gradient of B in A for 30 min (A: 0.1% FA; B: ACN, 0.1% FA) at a flow rate of 300 nl per min. Peptides were analysed in a nano ESI qQTOF mass spectrometer (6600 TripleTOF, AB Sciex). TripleTOF was operated in information-dependent acquisition mode, in which a 0.26 s TOF MS scan from 350–1250/*mz* was performed, followed by 0.05 s product ion scans from 100–1600/*mz* on the 50 most intense 2–5 charged ions. MS/MS information was sent to MASCOT v 2.3.02 or to PARAGON via v 4.5 (AB Sciex). The system sensitivity was controlled with 2 fmol of 6 proteins (LC Packings).

### ProteinPilot v4.5. search engine (ABSciex)

ProteinPilot default parameters were used to generate peak list directly from 6600 Triple TOFwiff files. The PARAGON algorithm of ProteinPilot v5.0 was used to search Expasy protein database (616,203 sequences; 181,334,896 residues) with the following parameters: trypsin specificity, cys-alkylation, no taxonomy restriction, and the search effort set to ‘through'. The special factor: protein phosphorylation was set and biological modifications were searched. To avoid using the same spectral evidence in more than one protein, the identified proteins were grouped based on MS/MS spectra by the Protein-Pilot Progroup algorithm. Thus, proteins sharing MS/MS spectra were grouped, regardless of the peptide sequence assigned. The protein within each group that explained more spectral data with confidence was shown as the primary protein of the group. Only the proteins of the group for which there was individual evidence (unique peptides with enough confidence) were listed, usually towards the end of the protein list.

### Nucleic acid purification and quantitative PCR analysis

Total RNA was extracted using TRIzol reagent and cDNA synthesized using SuperScript III reverse transcriptase kit (both from Invitrogen). cDNA products were amplified using and Applied Biosystems StepOne plus Fast Real-Time PCR System. Where indicated, Taqman probes were from Applied Biosystems. Sequences of the primers and Taqman probes used in this study are listed in Supplementary Tables 3 and 4, respectively.

### Statistical methods

The Student's *t*-test was used to estimate statistical significance between categories. Relative values (percentages) were normalized using arcsine transformation before carrying out their statistical comparison. Results are presented as mean±s.e.m. (standard error of the mean). At least three independent experiments (*n*) were carried out for statistical comparison. In addition, each quantitative PCR experiment (*n*) was carried out in triplicate.

## Additional information

**How to cite this article:** Prieto, J. *et al*. Early ERK1/2 activation promotes DRP1-dependent mitochondrial fission necessary for cell reprogramming. *Nat. Commun.* 7:11124 doi: 10.1038/ncomms11124 (2016).

## Supplementary Material

Supplementary Information Supplementary Figures 1-6, Supplementary Tables 1-4 and Supplementary References
