# Supplementary material for: Early ERK1/2 activation promotes DRP1-dependent mitochondrial fission necessary for cell reprogramming
Source: Nat Commun. 2016 Mar 31;7:11124. doi: 10.1038/ncomms11124 (PMC4821885; doi:10.1038/ncomms11124)
Supplement: Supplementary Information — Supplementary Figures 1-6, Supplementary Tables 1-4 and Supplementary References [file ncomms11124-s1.pdf]

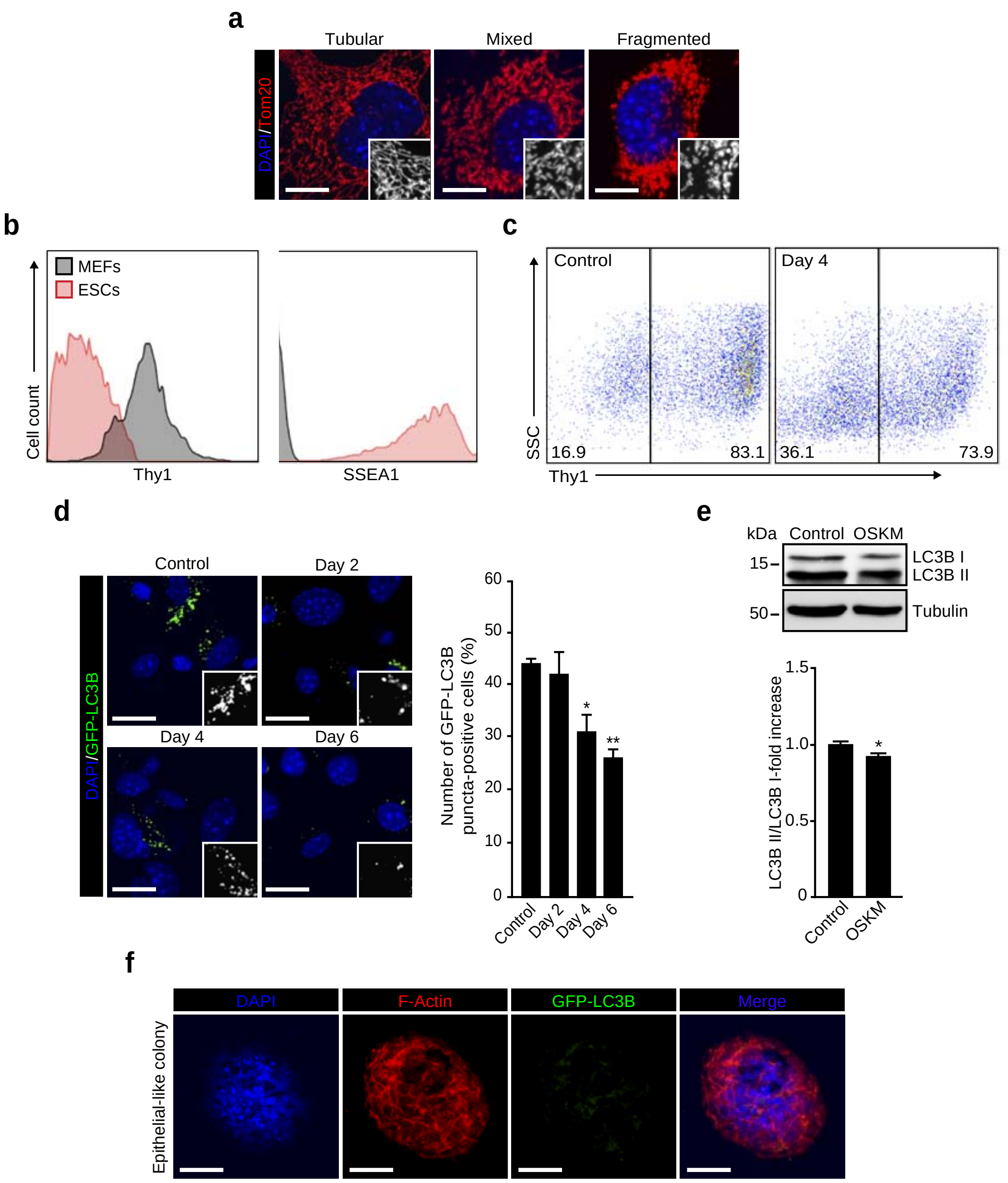

Supplementary Figure 1. Epithelial-like colonies are negative for autophagic markers. (**a**) Representative confocal images of wild type MEFs stained with anti-Tom20 antibody (red) illustrating the different mitochondrial morphologies observed in the cultures. Insets show a black and white magnification of the pictures. DAPI (blue) was used as a nuclear counterstaining. Scale bar, 12 µm. (**b**) Histograms showing the expression of the indicated markers in MEFs (gray) or ES cells (ESCs, red) assessed by flow cytometry. (**c**) Dot plots showing the expression of Thy1 in MEFs before (Control) or 4 days after OSKM expression. Percentages of the Thy1-negative and -positive are shown at the bottom of the dot plots. SSC, Side Scatter. (**d**) GFP-LC3B-expressing MEFs were mock- or OSKM-infected. At the

indicated days cells were treated during 4 hours with 50 μM Chloroquine and then processed for IF. Representative confocal images on the left show cells positive for GFP-LC3B puncta (green). Insets show a black and white magnification of the pictures. DAPI (blue) was used as a nuclear counterstaining. Scale bars, 24 μm. Graph on the right shows the quantification of cells positive for GFP-LC3B puncta before or at the indicated days after OSKM expression (n = 3). (**e**) Lysates of MEFs control or OSKM-infected were analysed by immunoblotting using the indicated antibodies. Graph on the right shows the quantification of the data (n = 3). (**f**) GFP-LC3B-expressing MEFs were mock- or OSKM-infected and eight days post-infection cells were treated during 4 hours with 50 μM Chloroquine and then processed for immunofluorescence with Alexa Fluor 555-Phalloidin (red). Representative confocal images of an epithelial-like colony showing the absence of GFP-LC3B puncta. DAPI (blue) was used as a nuclear counterstaining. Scale bar, 40 μm. Data are represented as mean ± s.e.m., and one-tailed unpaired Student's t-test was used to compare data sets (*$P < 0.05$; **$P < 0.01$; ***$P < 0.001$; ****$P < 0.0001$).

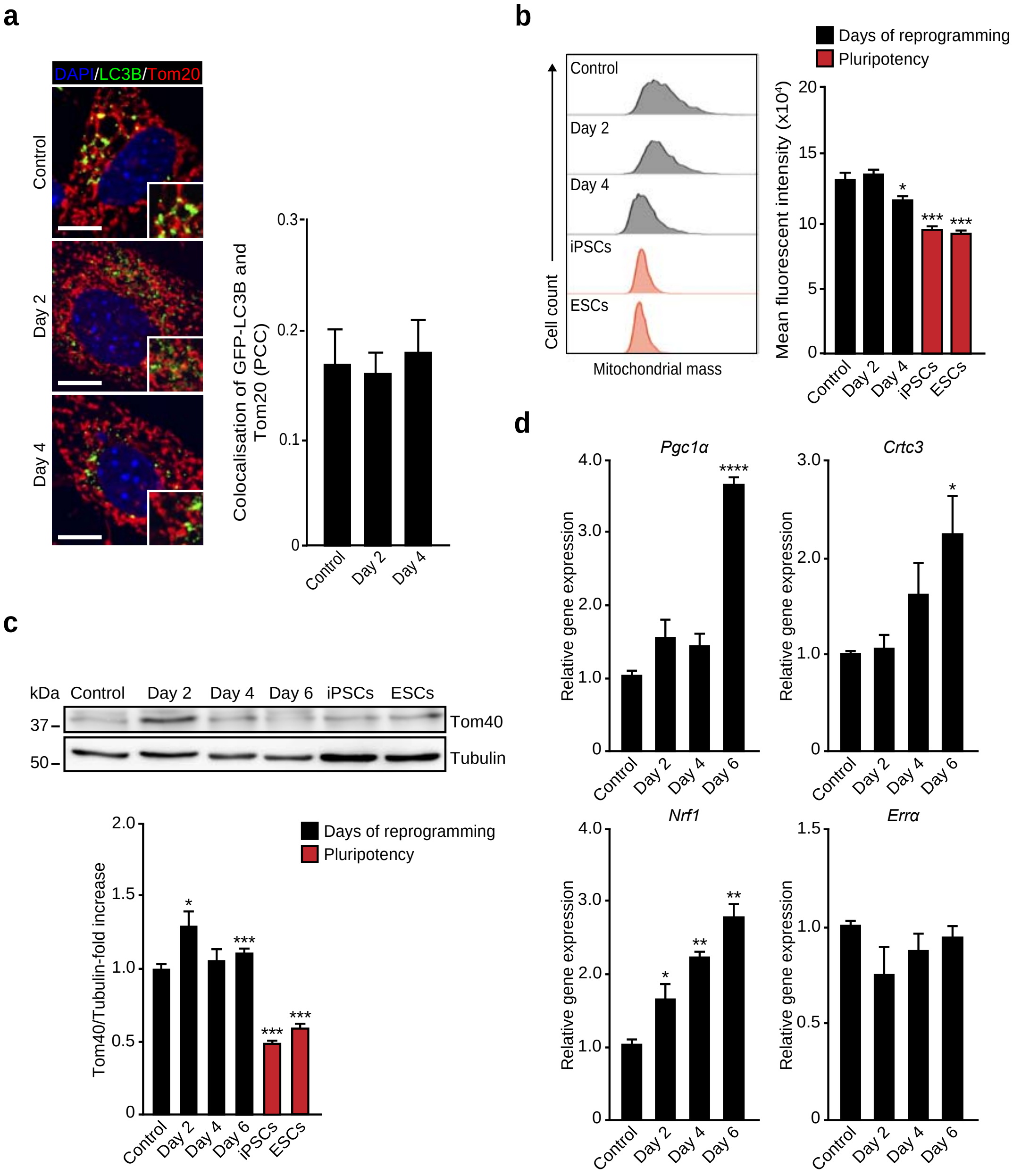

Supplementary Figure 2. Mitochondrial fission is not associated with mitophagy. (**a**) Representative confocal images of MEFs transduced and treated as in Supplementary Fig. 1d that were subjected to immunofluorescence analysis using anti-Tom20 antibody (red) for labelling mitochondria. Insets show a magnification of the pictures. DAPI (blue) was used as a nuclear counterstaining. Scale bars, 12 μm. Graph on the right shows the colocalisation of GFP-LC3B with Tom20 assessed by the Pearson correlation coefficient (PCC) of both stainings in the cells before (Control) or at the indicated days after OSKM expression (n = 3). (**b**) Representative flow cytometry histograms of MEFs stained with mitochondrial membrane potential-independent Mitotracker Green FM for assessing mitochondrial mass before (Control) or at the indicated days after transduction

with OSKM-encoding retroviruses. Pluripotent iPS and ES cells are showed as controls. Right graph shows the quantification of the Mean Fluorescence Intensity of the histograms shown in the left (n = 3). (**c**) Cells were treated as in (b) and total lysates subjected to immunoblotting analysis using the indicated antibodies to assess mitochondrial mass. Graph on the bottom shows the quantification of the data (n = 3). (**d**) Total RNA was extracted from mock-infected (control) or OSKM-infected wild type MEFs for the indicated days. The expression of the indicated genes implicated in mitochondrial biogenesis was then assessed by qPCR and represented as relative gene expression normalised to control MEFs (n = 3). Data are represented as mean ± s.e.m., and one-tailed unpaired Student's t-test was used to compare data sets (*$P < 0.05$; **$P < 0.01$; ***$P < 0.001$; ****$P < 0.0001$).

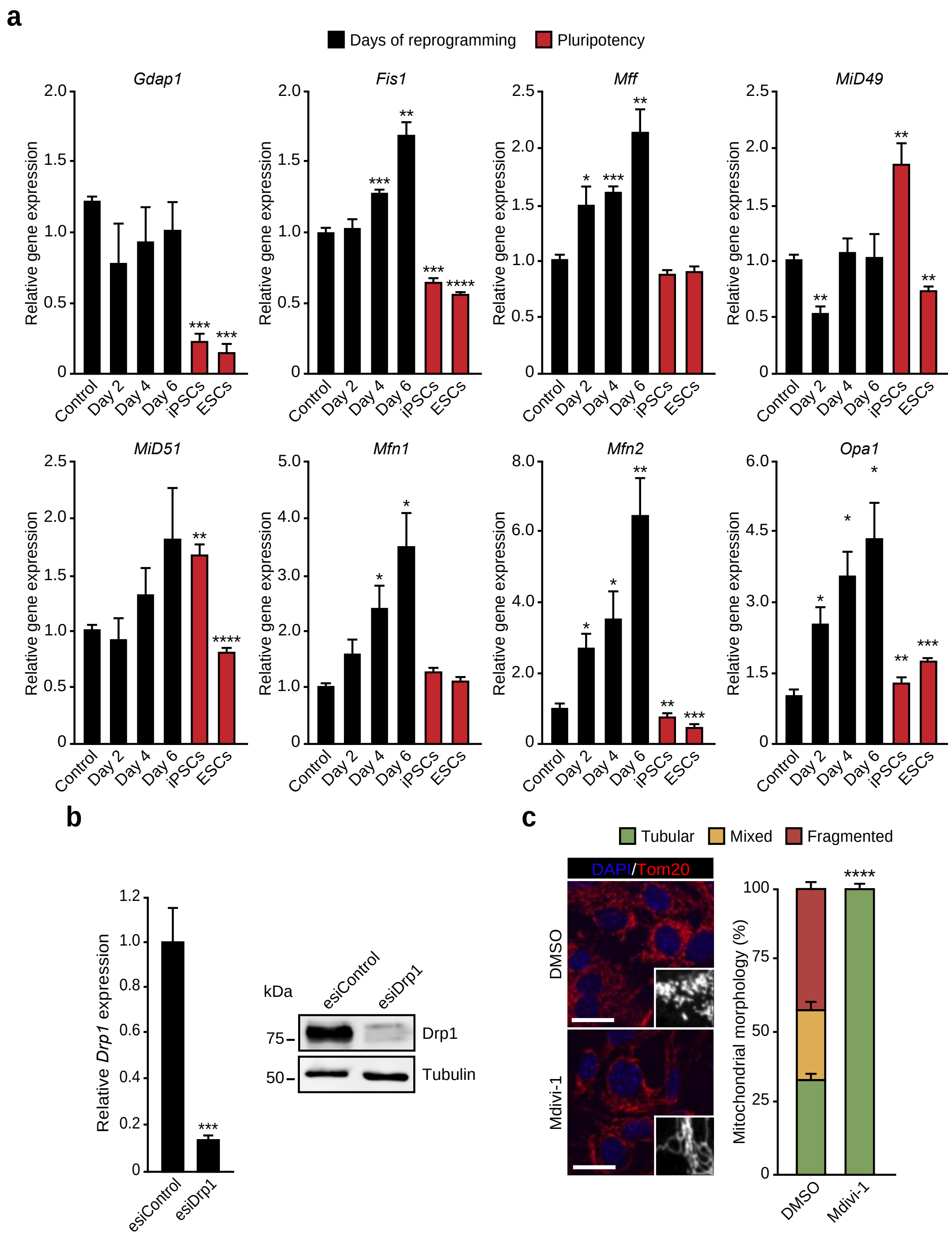

Supplementary Figure 3. Expression of mitochondrial dynamics regulatory factors. (**a**) Total RNA was extracted from mock- (Control) or OSKM-infected wild type MEFs for four days (black bars), or from the indicated pluripotent cells (red bars). The expression of the indicated genes was then assessed by qPCR and represented as relative gene expression normalised to control MEFs (n = 3). (**b**) Graph on the left shows the expression of *Drp1* assessed by qPCR in MEFs four days after being transduced with the OSKM factors in presence of esiRNAs

targeting GFP as control (esiControl) or mouse *Drp1* (esiDrp1) (n = 3). Panels on the right show the assessment of Drp1 and Tubulin proteins expression by immunoblotting using specific antibodies in cells treated as before. (**c**) Representative confocal images of MEFs expressing the reprograming factors during four days in the presence of Mdivi-1 (50 μM) and stained with anti-Tom20 antibody (red) to assess the indicated mitochondrial morphologies. Insets show a black and white magnification of the pictures. DAPI (blue) was used as a nuclear counterstaining. Scale bars, 24 μm. Graphs on the right show the quantification of the indicated mitochondrial morphologies observed in cells treated as above (n = 3). Data are represented as mean ± s.e.m., and one-tailed unpaired Student's t-test was used to compare data sets (**P* < 0.05; ***P* < 0.01; ****P* < 0.001; *****P* < 0.0001).

a

Protein identification

Fraction	Identified protein	Unused	%Cov	%Cov(95)	Nº Peptides (95%)
Cytosolic	Dynamin-1-like protein (DNM1L)	405,6	97,8299975	86,8200	823
Mitochondrial	Dynamin-1-like protein (DNM1L)	105,39	81,129998	72,1000	115

Peptide modifications

Fraction	Modified residue	Sequence	Confidence	dMass
Mitochondrial	pSer579	SKPIPIMPASPQK	99,00	0,00369
Cytosolic	pSer330, pThr338	DDKSATLLQLITKFATEYCNTIEGTAK	97,94	0,00737
Cytosolic	pSer647	DTLQSELVGQLYKSSLLDDLLTESEDMAQR	90,21	0,05967
Cytosolic	pSer175	FISNPNSIILAVTAANTDMATSEALK	99,00	0,03330
Cytosolic	pSer136	GVSPEPIHLKIFSPNVVNLTLVDLPGMTK	99,00	0,03157
Cytosolic	pSer450	IIQHCSNYSTQELLR	99,00	0,02359
Cytosolic	pThr479	LPVTNEMVHNLVAIELAYINTK	99,00	0,03727
Cytosolic	pSer71, pThr78	QLVHVSQEDKRKTTGEENGVEAEEWGK	99,00	-0,01278
Cytosolic	pSer175	RFISNPNSIILAVTAANTDMATSEALK	99,00	0,03811
Cytosolic	pSer71	RPLILQLVHVSQEDK	99,00	0,00315
Cytosolic	pSer579	SKPIPIMPASPQKGHAVNLLDVPVPVAR	99,00	0,00072
Cytosolic	pSer579	SKPIPIMPASPQK	99,00	0,00696
Cytosolic	pSer136	SPEPIHLKIFSPNVVNLTLVDLPGMTK	99,00	0,05696
Cytosolic	pSer656	SSLLDDLLTESEDMAQR	99,00	0,03261
Cytosolic	pSer616	SYFLIVR	99,00	0,01953
Cytosolic	pSer656, pSer657	VGQLYKSSLLDDLLTESEDMAQR	97,38	0,07306

b

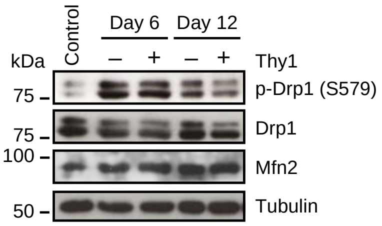

Supplementary Figure 4. Drp1 post-translational modifications in ES cells. (**a**) Summary of the phosphorylated residues found in cytosolic or mitochondria-associated Drp1 in ES cells by LC-MS/MS. Upper table shows the protein identification in both fractions sorted by Unused ProtScore. Unused, assessment of the protein confidence parameter for the indicated protein, calculated from the peptide confidence for peptides from the spectra that were not completely "used" already by the higher scoring-winning proteins; %Cov (coverage), percentage of matching amino acids from identified peptides, which showed a confidence greater than 0, divided by the total number of amino acids in the sequence; %Cov(95%), the percentage of matching amino acids from identified proteins that showed a

confidence ≥ 95%, divided by the total number of amino acids in the sequence. Lower table shows the details of the identified peptides in both subcellular fractions. Confidence, the confidence for the peptide identification, expressed as percentage; dMass, the mass difference between molecular weight of the precursor and the theoretical molecular weight of the matching peptide sequence. (**b**) MEFs were transduced, sorted as in Figure 2b into Thy1-positive and -negative cell populations at day 4 post-transduction and cultured in iPS cell medium. At the indicated days, total lysates from the different cell populations were subjected to immunoblotting analysis using the indicated antibodies. A representative immunoblot out of two independent experiments is shown.

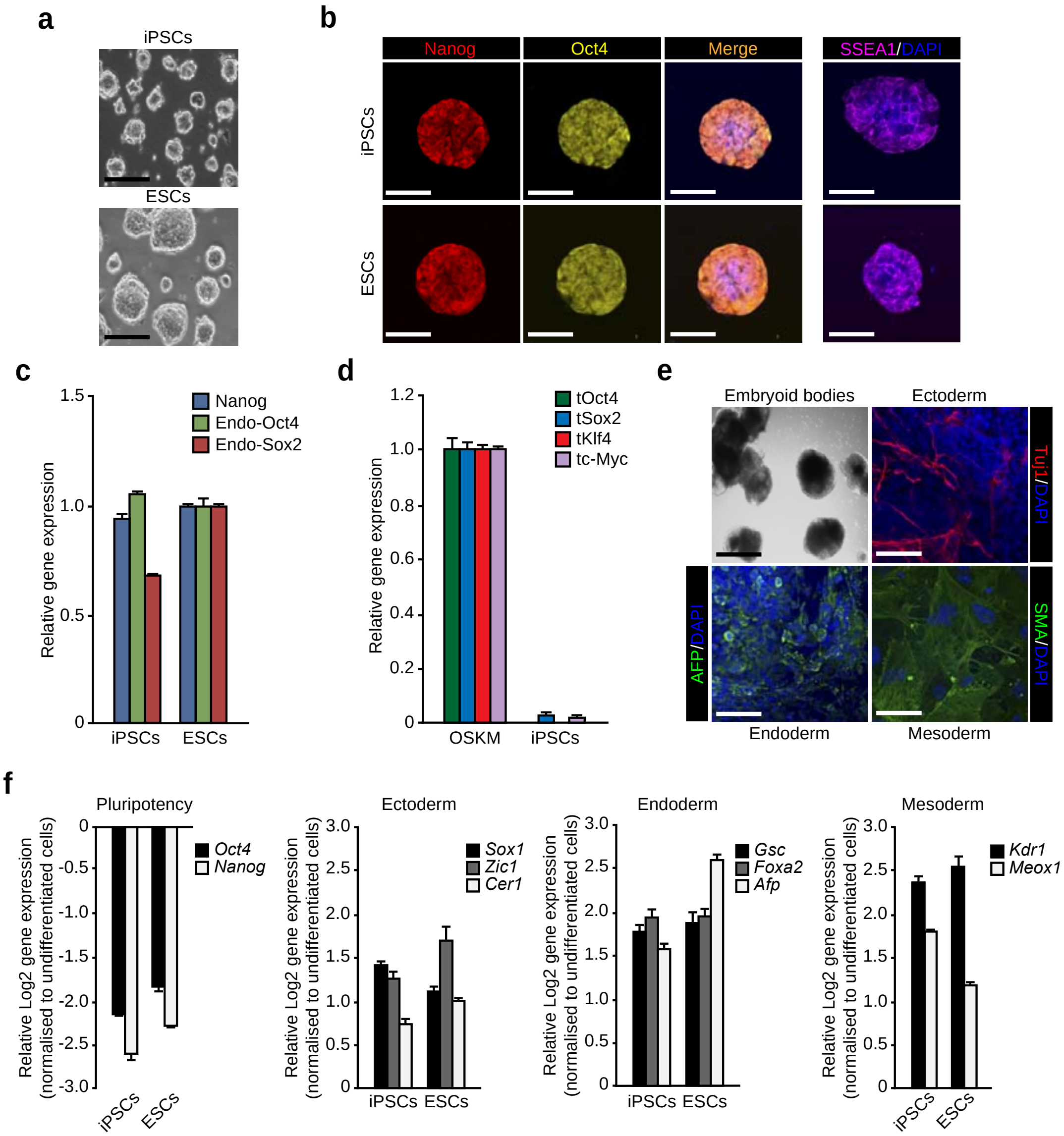

Supplementary Figure 5. Molecular and functional characterisation of wild type iPS cells. (**a**) A representative iPS cell clone (iPSCs) or the E14Tg2a ES cell line (ESCs) were photographed. Scale bar, 130 µm. (**b**) Pluripotent cells were subjected to IF staining with the indicated antibodies, using DAPI (blue) as a nuclear counterstaining. Scale bar, 30 µm. IF images show different fields compared with phase pictures. (**c, d**) Total RNA was extracted from the indicated pluripotent cells and expression of (**c**) pluripotency markers or (**d**) exogenous OSKM cDNAs was assessed by qPCR and represented as relative gene expression normalised to undifferentiated ES cells in (**c**) or OSKM-infected cells (OSKM) in (**d**). (**e**) iPS cell clone was subjected to EB differentiation and photographed 7 days after (top panel). Scale bar, 500 µm. EBs were then seeded and 2 days later cells were subjected to IF with the indicated antibodies, using DAPI (blue) as a nuclear counterstaining (lower panels). Scale bar, 40 µm. (**f**) Total RNA was extracted from 7 days old EBs and the expression of pluripotency, ectoderm, endoderm or

mesoderm markers was assessed by qPCR and represented as relative gene expression normalised to undifferentiated ES cells. Data are represented as mean ± s.e.m. (n = 3).

Prieto et al., Supplementary Figure 6

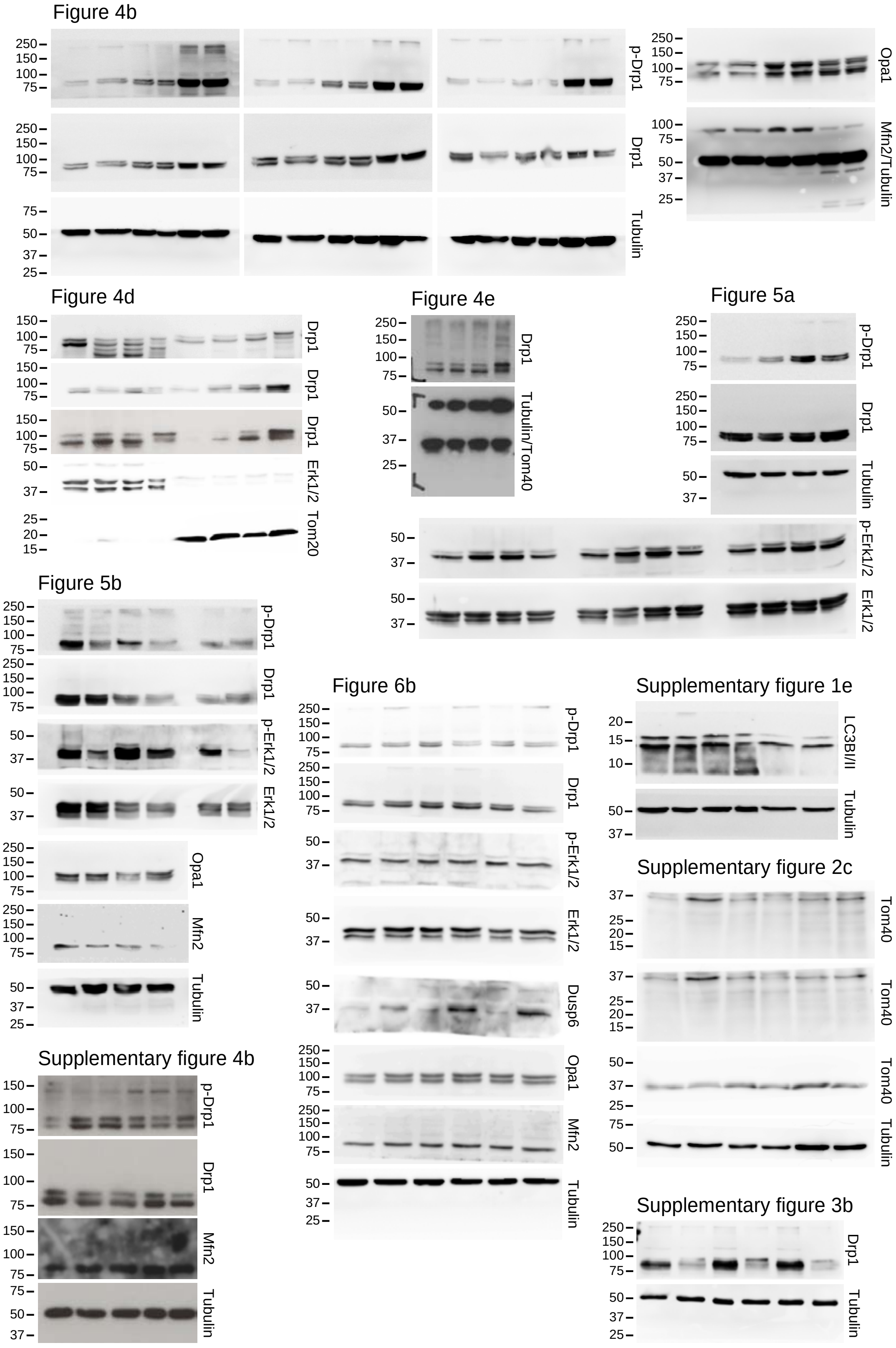

Supplementary Figure 6. Uncropped blots.

SUPPLEMENTARY TABLES

Prieto et al., Supplementary Table 1

Antigen	Host	Company	Reference	Dilution and applications
Alpha-fetoprotein	Rabbit	Abcam	ab46799	1:100 (IF)
α-Tubulin	Mouse	SCBT	sc-32293	1:5000 (WB)
βIII-Tubulin (Tuj1)	Rabbit	Covance	MRB-435P	1:1000 (IF)
Drp1	Rabbit	CST	#8570	1:1000 (WB); 1/50 (IF)
p-Drp1 (Ser616)	Rabbit	CST	#3455	1:1000 (WB)
Erk1/2	Rabbit	CST	#9102	1:1000 (WB)
p-Erk1/2	Mouse	CST	#9106	1:2000 (WB)
Dusp6	Rabbit	Dr. R. Pulido Lab	in-house	1:1000 (WB)
LC3B	Rabbit	CST	#2775	1:1000 (WB)
Mfn2	Rabbit	Sigma-Aldrich	AV42420	1:1000 (WB)
Nanog	Rabbit	Cosmo Bio Co.	RCAB0001P	1:100 (IF); 1:1000 (WB)
Oct4	Mouse	SCBT	sc-5279	1:50 (IF)
Opa1	Mouse	BD	# 612606	1:1000 (WB)
Smooth Muscle Actin	Mouse	Abcam	ab7817	1:100 (IF)
SSEA1	Mouse	SCBT	sc-21702	1:10 (IF and FC)
Tom-20	Rabbit	SCBT	sc-11415	1:50 (IF); 1:500 (WB)
Tom-40	Mouse	SCBT	sc-365467	1:500 (WB)
Thy1 (CD90.2)	Rat	eBioescience	14-0903-81	1:400 (IF, FC)
Rat IgG2b K Isotype Control	Rat	eBioescience	16-4031-81	1:400 (FC)

Supplementary Table 1. Primary antibodies used in this study. SCBT, Santa Cruz Biotechnology; CST, Cell Signaling Technologies; BD, BD Biosciences; IF, immunofluorescence; FC, flow cytometry; WB, western blot.

Target	Host	Conjugate	Company	References	Dilution and applications
Mouse IgG	Donkey	AF®488	TF	A-21202	1:1000 (IF)
Mouse IgG	Donkey	AF®555	TF	A-31570	1:1000 (IF)
Mouse IgG	Donkey	AF®647	TF	A-31571	1:1000 (IF)
Mouse IgM	Donkey	AF®555	TF	A-21426	1:1000 (IF)
Rabbit IgG	Donkey	AF®488	TF	A-21206	1:1000 (IF)
Rabbit IgG	Donkey	AF®555	TF	A-31572	1:1000 (IF)
Rabbit IgG	Donkey	AF®647	TF	A-31573	1:1000 (IF)
Rat IgG	Donkey	AF®488	TF	A-11006	1:1000 (IF, FC)
Mouse IgG	Goat	HRP	TF	31432	1:5000 (WB)
Rabbit IgG	Goat	HRP	TF	31460	1:5000 (WB)

Supplementary Table 2. Secondary antibodies used in this study. TF, Thermo Fisher Scientific; SCBT, Santa Cruz Biotechnologies; AF®, AlexaFluor®; HRP, horseradish peroxidase; IF, immunofluorescence; WB, western blot; FC, flow cytometry.

Gene	Forward primer (5' to 3')	Reverse primer (5' to 3')
Crtc3	TGACTCACCTGGGGATAAGAAC	GTGGCACTTGAGGGACGAG
Drp1	CAGGAATTGTTACGGTTCCCTAA	CCTGAATTAACTTGTCCCGTGA
Dusp6	ATGATGAGGTCTTCAGTCTC	CAAAATACCCCTTGAGACAC
Errα	TTCGGCGACTGCAAGCTC	CACAGCCTCAGCATCTTCAATG
Fis1	TGTCCAAGAGCACGCAATTTG	CCTCGCACATACTTTAGAGCCTT
Gapdh	GCACAGTCAAGGCCGAGAAT	GCCTTCTCCATGGTGGTGAA
Gdap1	TCCTTCAGCTCTCAAAAGGTGC	GCGCATAAACCAAGGCTCATT
Mff	ATGCCAGTGTGATAATGCAAGT	CTCGGCTCTCTTCGCTTTG
Mid49	AGCCCACGCCCATTCATTC	TGGAGCCCGTCGTAGAGAG
Mid51	GGTGAGCGCAAAGGGAAGA	AATGCCCAACATAGCTGCTCC
Mfn1	ATGGCAGAAACGGTATCTCCA	CTCGGATGCTATTCGATCAAGTT
Mfn2	TGACCTGAATTGTGACAAGCTG	AGACTGACTGCCGTATCTGGT
Nrf1	CCACATTACAGGGCGGTGAA	AGTGGCTCCCTGTTGCATCT
Opa1	ACAGCAAATTCAAGAGCACGA	TTGCGCTTCTGTTGGGCAT
Pgc1α	CGGAAATCATATCCAACCAG	TGAGGACCGCTAGCAAGTTTG

Supplementary Table 3. Oligonucleotides used in this study.

Gene	Catalogue no./Sequence	Assay
Afp	Mm00431715_m1	TaqMan gene expression assay
Cer1	Mm03024044_m1	TaqMan gene expression assay
Foxa2	Mm00839704_mH	TaqMan gene expression assay
Gapdh	Mm99999915_g1	TaqMan gene expression assay
Gsc	Mm00650681_g1	TaqMan gene expression assay
Kdr1	Mm01222421_m1	TaqMan gene expression assay
Meox1	Mm00440285_m1	TaqMan gene expression assay
Nanog	Mm02384862_g1	TaqMan gene expression assay
Oct4	Mm00658129_gH	TaqMan gene expression assay
Sox1	Mm00486299_s1	TaqMan gene expression assay
Sox2	Mm03053810_S1	TaqMan gene expression assay
Zic1	Mm00656094_m1	TaqMan gene expression assay
tOct4-F	TGGTACGGGAAATCACAAGTTTGTA	Custom TaqMan gene expression assay for retroviral transgene[1]
tOct4-R	GGTGAGAAGGCGAAGTCTGAAG	
tOct4-probe	FAM-CACCTTCCCCATGGCTG-MGB	
tSox2-F	TGGTACGGGAAATCACAAGTTTGTA	Custom TaqMan gene expression assay for retroviral transgene[1]
tSox2-R	GCCCGGCGGCTTCA	
tSox2-probe	FAM-CTCCGTCTCCATCATGTTAT-MGB	
tKlf4-F	TGGTACGGGAAATCACAAGTTTGTA	Custom TaqMan gene expression assay for retroviral transgene[1]
tKlf4-R	GAGCAGAGCGTCGCTGA	
tKlf4-probe	FAM-CCCCTTCACCATGGCTG-MGB	
tc-Myc-F	TGGTACGGGAAATCACAAGTTTGTA	Custom TaqMan gene expression assay for retroviral transgene[1]
tc-Myc-R	GGTCATAGTTCCTGTTGGTGAAGTT	
tc-Myc-probe	FAM-CCCTTCACCATGCCCC-MGB	

Supplementary Table 4. Taqman probes used in this study. F, forward primer; R, reverse primer; probe, FAM-labelled fluorogenic probe designed for each transgene[1]. t-*geneX*, reprogramming factor cloned in pMX retroviral vector.

SUPPLEMENTARY REFERENCES

1. Theunissen T.W. *et al*. Nanog overcomes reprogramming barriers and induces pluripotency in minimal conditions. *Curr. Biol*. **21**, 65-71 (2011).
